# Pterygopalatine disjunction-associated SARME and the nasal cavity - A systematic literature review

**DOI:** 10.4317/jced.62026

**Published:** 2024-09-01

**Authors:** José Rômulo de Medeiros, Fábio Wildson Gurgel Costa, Francisco Samuel Rodrigues Carvalho, Ana Ericka de Araújo Mouta, Marcelo Ferraro Bezerra, José Moacir Marques da Costa Junior, Paulo Goberlânio de Barros Silva, Eduardo Costa Studart Soares

**Affiliations:** 1 Postgraduate Student, Division of Oral Maxillofacial Surgery, Postgraduate Program in Dentistry, Federal University of Ceará, Fortaleza, Ceará, Brazil. Professor Division of Oral and Maxillofacial Surgery, Fortaleza University (UNIFOR), Fortaleza, Ceará, Brazil; 2 Adjunct Professor, Division of Radiology, Postgraduate Program in Dentistry, Federal University of Ceará, Fortaleza, Ceará, Brazil; 3 Professor, Division of Oral and Maxillofacial Surgery, Federal University of Ceará – campus Sobral, Sobral, Ceará, Brazil; 4 Postgraduate student in Health Sciences, Federal University of Ceará (UFC), Fortaleza, Ceará, Brazil.; 5 Private Practioner, Ceará, Fortaleza, Brazil; 6 Professor, Division of Oral Pathology, Postgraduate Program in Dentistry, Unichristus University Center, Fortaleza, Ceará, Brazil; 7 Full Professor Titular, Division of Oral Maxillofacial Sugery, Postgraduate Program in Dentistry, Federal University of Ceará, Fortaleza, Ceará, Brazil

## Abstract

**Background:**

This study aimed to evaluate the effect of surgically assisted rapid maxillary expansion (SARME) with pterygopalatine disjunction (PD) on the nasomaxillary complex structures.

**Material and Methods:**

A systematic two-phase review, recorded in the PROSPERO database, was conducted. Search strategies were performed using PubMed, Scopus, Web of Science, COCHRANE, LILACS and DOSS databases, including gray literature (Open Grey, Google Scholar, and ProQuest). The methodological quality and evidence of the included studies were assessed.

**Results:**

Out of 1017 studies, 10 met the inclusion criteria. Generally, a moderate risk of bias was noted. The studies involved 236 adults evaluated preoperatively and postoperatively. Key outcomes assessed included nasal cavity volume, minimum cross-sectional area (MCSA), nasal septum positioning, nasal cavity width, and nose volume (soft tissues).

**Conclusions:**

Although findings indicated increased MCSA, nasal cavity, and nasopharynx volumes, and no changes in nasal septum post-SARME + PD, the current evidence is insufficient for definitive clinical recommendations due to study limitations, particularly the small number of included studies. More clinical studies with robust methodologies and low risk of bias are needed.

** Key words:**Nasal septum, nasal cavity, palatine expansion technique.

## Introduction

Transverse changes represent the most common skeletal deformities in the oral-maxillofacial complex (1), occurring independently or in conjunction with other abnormalities (2). Maxillary transverse hypodevelopment is characterized by a high-arched palate, tooth crowding, rotations, and unilateral or bilateral posterior crossbite, often accompanied by a deficiency in arch perimeter. It is frequently associated with nasal respiratory issues, adenoid hypertrophy, oral respiration, and middle ear diseases (3,4).

Decreased distance between nasal cavity walls and the septum increases nasal airflow resistance, complicating nasal breathing (5). Nasal respiration is vital for stomatognathic balance. Skeletally mature individuals with discrepancies exceeding 5 mm may require surgically assisted rapid maxillary expansion (SARME) (1-4). This surgical technique is known for its predictability, achieving sufficient expansion, and maintaining long-term stability (6).

Although SARME + PD is commonly performed, its effects on the nasal cavity, septum, and paranasal region remain inadequately understood. Some studies suggest benefits such as increased expansion in the posterior palate/nasal cavity floor (12) and significant volume increases in the nasopharynx and oropharynx (11). However, no systematic review has comprehensively correlated SARME with or without PD to changes in the nasal cavity and nasomaxillary complex. Therefore, this review aims to assess alterations in the nasal cavity, septum, and paranasal region following SARME + PD, addressing the question: “Do patients with transverse deficiency undergoing SARME + PD exhibit structural changes in the nasal cavity?”

## Material and Methods

-Protocol and registration

This systematic review followed the Preferred Reporting Items for Systematic Reviews and Meta-Analyses (PRISMA) guidelines and was registered in PROSPERO (International Registry of Systematic Reviews; identifier CRD42020133208).

-Eligibility criteria

Inclusion criteria: Clinical trials and observational studies evaluating SARME + PD; assessment of changes in the nasal cavity or nasomaxillary complex, including bone and cartilaginous structures; evaluations using radiographic examinations, helical or cone-beam CT, AR, rhinomanometry, frontal cephalometry, and photographs in studies with preoperative and postoperative assessments. Exclusion criteria: Reviews, letters to the editor, personal opinions, book chapters, scientific event summaries, SARME in individuals without skeletal maturity, studies with multiple treatment modalities, patients with syndromes, craniofacial anomalies, or systemic deficiencies, lack of data on nasal cavity or nasomaxillary complex changes, absence of pterygoid disjunction, studies not in Latin (Roman) alphabet, duplicate samples, and articles unavailable for full reading despite author contact.

-Sources of information

Detailed search strategies tailored to each database—PubMed, Scopus, Web of Science, COCHRANE, LILACS, and DOSS—were implemented. Gray literature sources were also included, encompassing the first 50 most relevant articles from Google Scholar, OpenGrey, and ProQuest. The search covered articles published up to May 31, 2024, without time restrictions. Additional articles were identified through manual searches.

-Search

Appropriate search terms and truncation were selected for each database query. Further information on the search strategies is available in Appendix A (supplementary data). Retrieved references were managed using EndNote X8® software (Thomson Reuters, New York, NY) to eliminate duplicates.

-Selection of studies

Phase 1 involved two reviewers (JRM and AEAM) independently screening titles and abstracts using the Rayyan® application for systematic reviews (Qatar Computing Research Institute, Doha, Qatar). Phase 2 consisted of the same reviewers independently applying inclusion criteria to the full texts. A third examiner (FSRC) critically assessed the reference lists of selected studies. Disagreements were resolved through discussion between the two primary reviewers. In cases of unresolved disagreements, the third and fourth authors (FSRC and ECSS) participated in the final decision-making process.

-Data collection process

One author (JRM) extracted data from the selected studies, which was then cross-checked by a second author (FSRC). Disagreements between them were resolved through discussion. If consensus could not be reached, a third author (ECSS) made the final decision.

-Information collected

The following data were recorded: year of publication, origin, study design, participants (sample size, sex, age), interventions (surgical technique, distractor type), measurement periods, anesthesia type, distraction rate, presence of control group, clinical outcomes evaluated, latency period, total distraction amount (mean), main nasal findings, and primary nasal measurements.

-Risk of bias (RoB) in individual studies

The Meta-Analysis of Statistics Assessment and Review Instrument tool assessed the Risk of Bias (RoB) in the included studies. RoB was categorized as “high” (when studies had a “yes” percentage less than 49%), “moderate” (when studies scored between 50% and 69%), and “low” (when studies scored 70% or higher) based on similar methodologies. The RevMan software (Review Manager, version 5.3, Cochrane Collaboration, Copenhagen, Denmark) was used to generate a summary of the RoB.

-Certain of evidence

The certainty of evidence was assessed using the Grading of Recommendations, Assessment, Development and Evaluation (GRADE) approach, which evaluates how confident we can be that an estimate of effect or association reflects the true effect (http://gdt.guidelinedevelopment.org).

## Results

-Study selection

A total of 1017 articles were retrieved from six electronic databases. After removing duplicates, 538 articles underwent title and abstract screening, resulting in 70 potentially relevant studies selected for full-text reading. No articles were found through gray literature searches (Google Scholar, ProQuest, and OpenGrey), but three additional articles were identified through manual search. Ultimately, 10 studies met the inclusion criteria and were included in this systematic review.

-Characteristics of the studies

The studies included participants from Brazil, Netherlands, USA, Turkey, Poland, and Iran, totaling 190 participants (62 males and 28 females). The mean ages ranged from 18.8 years (13) to 28.6 years (12). All participants underwent SARME + PD, with general anesthesia administered in five studies (7-10). One study used general anesthesia for eight patients and local anesthesia for one patient (12); two studies did not specify the type of anesthesia used (13,14).

The distraction devices varied across studies: Hyrax (7,8,13-15), Hyrax and Haas (9), Hyrax and transpalatal distractor (TPD) (11), and TPD (16); one study did not specify the device used (12). Additionally, methodologies for evaluating outcomes varied among the studies: linear, area, or angular measurements using CT (7,12-14); volumetric and area measurements using CT (8); volumetric measurements using CT and photographs (11); volumetric and area measurements using augmented reality (AR) (10); and AR combined with frontal cephalometry (9).

Only two studies (7,8) conducted assessments at three time points: T0 (installation of the expander device), T1 (immediately after active expansion), and T3 (approximately 6 months post-expansion). The remaining studies performed two evaluations: immediately after active expansion and at intervals ranging from 3 months (13) to 22 ± 7 months (11) post-expansion.

-RoB in individual studies

**Figure 1:**
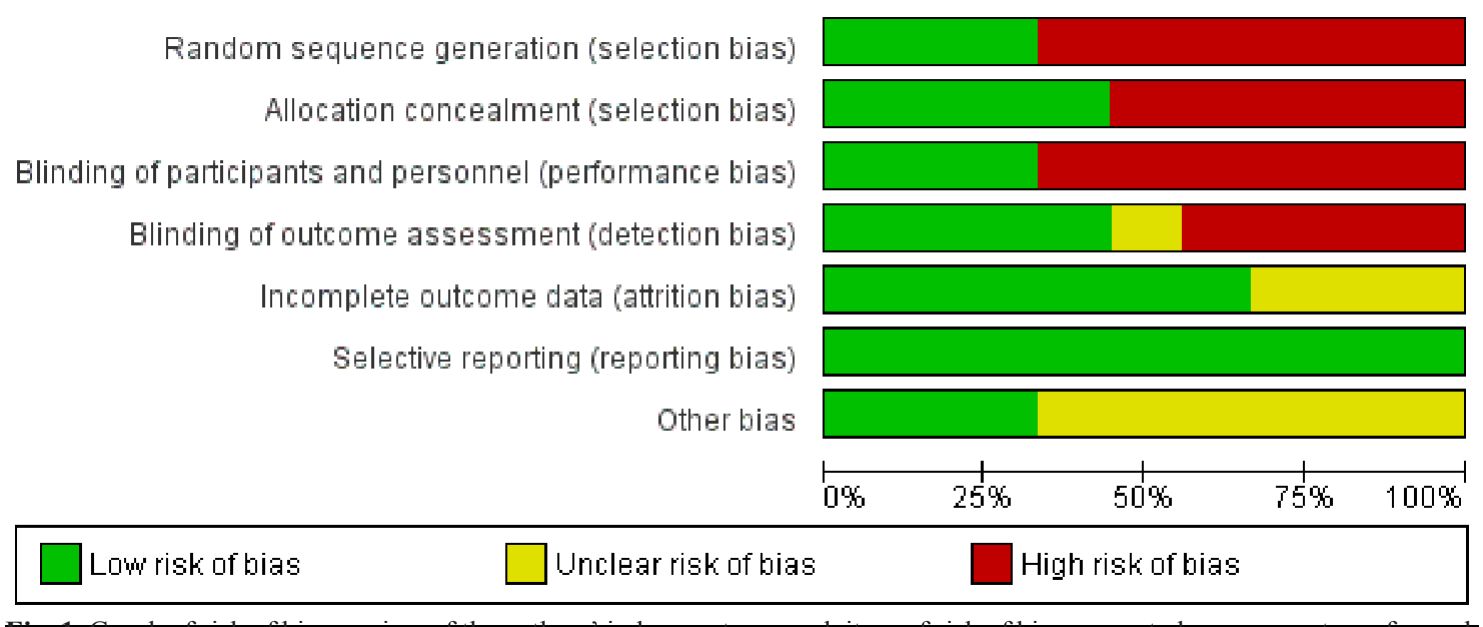
Graph of risk of bias: review of the authors’ judgements on each item of risk of bias presented as a percentage, for each study included.

In a comprehensive analysis, the included studies exhibited moderate risk of bias (RoB), with 37.5% classified as low RoB, 25% as moderate, and 37.5% as high (Fig. 1). The studies primarily demonstrated high RoB, particularly concerning sample size and blinding.

-Summary of results

Among the studies evaluating nasal cavity volume, two reported a statistically significant increase after SARME + PD (10,11), while one did not find such an increase (8). Regarding minimum cross-sectional area (MCSA), two studies showed a statistically significant increase (9,10). One study observed a statistically significant increase in nasal volume within soft tissues (11). Studies measuring linear dimensions of the nasal cavity floor reported increases following SARME + PD (7,13,14). However, the single study assessing changes in nasal septum position after SARME + PD found no significant alteration (12).

-Additional analysis and confidence in the cumulative evidence

Based on the GRADE criteria for assessing evidence quality and recommendation strength in health decision-making, certainty regarding the absence of nasal volume increase after SARME + PD was rated as high in one study (8), and very low in two others (10,11). Regarding nasal floor enlargement after SARME + PD, certainty was high in one study (7), but low in two others (13,14).

## Discussion

Despite its widespread use in oral and maxillofacial surgery, systematic reviews continue to explore SARME, with and without PD, and its effects on the oral-maxillofacial complex. These reviews aim to improve understanding of upper airway implications (17) and the utility of cone-beam CT for SARME outcomes (18). Another review assessed expansion outcomes in the anterior and posterior maxillary regions and SARME complications (19). Recently, a review examined SARME’s impact on mandibular positioning with and without PD (20).

Reaching precise conclusions is challenging due to varied study designs and non-standardized measurement methods. Generally, studies indicate increased nasal volume after SARME + PD, but comparing AR and CT findings is difficult. An influential AR study reported increased nasal volume following SARME - PD (21), and another AR study in eight adults undergoing SARME without PD showed significant increases in nasal cavity volume and nasal valve area due to anterior and posterior MCSA expansion (22). Conversely, a recent randomized clinical trial using CT found no significant increase in nasal cavity volume after SARME + PD (8). Thus, more randomized trials comparing SARME + PD and SARME - PD using both AR and CT within the same patient cohort are needed.

Some studies have reported improved nasal breathing using subjective questionnaires (23,24). The NOSE scale was used in a study with SARME + PD patients, showing either improvement or no worsening of nasal obstruction based on preoperative and 6-month follow-up scores (25). The relationship between increased nasal cavity volume, airspace, and improved breathing, as well as the role of PD, remains unclear (12). Schwarz *et al*. (12) attributed increased nasal airway size to maxillary rotational expansion, reduced palatine shelf rotation, and reduced nasal mucosa inflammation, although they did not specify how inflammation levels were measured pre- and post-operatively. Koudstaal *et al*. (26) and Wriedt *et al*. (27) found that expansions in the anterior nasal cavity correlate with improved nasal breathing.

Both selected studies using AR on the MCSA of the nasal cavity reported a statistically significant increase after SARME + PD (9,10). However, neither study included a control group or a group without PD for comparison. AR measurements consider the nasal mucosa surface, which can be influenced by pre- or postoperative inflammation, affecting perceived bone expansion in nasal cavity walls and septum. This review found no studies using tomography for this outcome, limiting certainty on whether MCSA increased due to reduced mucosal inflammation or actual bone expansion. Additionally, the certainty regarding PD’s role in MCSA increase was very low. The use of nasal decongestants also complicates interpretation; Mitsuda *et al*. (10) noted higher MCSA values in the decongestant group.

This study reviewed existing literature on nasal septum changes, particularly concerning the impact of osteotomy of the nasal cavity floor and PD’s potential effect on septal displacement, especially in the posterior region. Only one selected study examined septal changes (12) without assessing PD’s role. The authors found no significant septal changes post SARME + PD, suggesting that PD facilitates uniform septal movement, not confined to the anterior region alone.

Only one study assessed changes in nose soft tissue volume and alar base dimensions, reporting statistically significant increases in both following SARME + PD ( Table 1), which has aesthetic implications for clinicians (11). Further studies are crucial to predict individual aesthetic impacts of varying expansion levels post-SARME. Regarding PD, it might facilitate more symmetric nostril changes and potentially more predictable aesthetic outcomes, but definitive studies are needed to confirm this hypothesis.

Regarding patient group distribution based on whether PD was performed, few studies (7,8,14) investigated nasal cavity and septum changes post-SARME. Only Baraldi *et al*. (9) compared SARME + PD with a control group without PD, finding that including PD did not significantly increase nasal cavity volume changes (24).

This systematic review showed minimal variation in the types of devices used, predominantly the Hyrax (tooth-borne) in seven out of eight studies. One study compared Hyrax with TPD and found no statistically significant difference in bone expansion achieved.

An often-overlooked factor impacting nasal cavity and septum findings is the extent of expansion achieved by distractor devices. Larger expansions are expected to have more significant effects on the facial skeleton.  Table 2 shows that among eight selected articles, only two mention total expansion achieved (7,14). Due to variability in reported expansion amounts, conclusive statements about SARME + PD’s impact on nasal structures are challenging.

Methodological limitations were evident in this systematic review, notably due to the small number of eligible studies, with only eight selected. Consequently, only qualitative analysis was feasible. Despite recognizing the potential benefits of a meta-analysis, the heterogeneity in outcome measurement methodologies across studies precluded this approach. Therefore, the scientific evidence quality could not be enhanced through meta-analysis in this study.

## Conclusions

This systematic review lacks sufficient evidence to conclusively assert that SARME + PD significantly influences nasal cavity, nasal septum, and nasal soft tissue structures. More rigorous randomized clinical trials are essential, comparing SARME − PD and SARME + PD groups. These studies should standardize distractor devices, measure maximum expansion achieved in both groups, and utilize both CT and AR methods for precise measurements. These steps are crucial for gaining clearer insights into the effects of SARME + PD on nasal structures.

## Figures and Tables

**Table 1:** General characterization of the studies included.

Authors	Origin	Study design	Participants (n)	Intervention	Control group	Outcome assessment	Primary outcome	Secondary outcomes
Baraldi et al., 2007	Brazil	Prospective controlled clinical trial	23 (8 male and 15 female), with 13 in the study group (mean age = 26.10 ± 6.85; 6 female and 4 male)	SARME + PD	Yes	AR and frontal cephalometric radiography	There was no difference between the groups in relation to nasal volumes. There was a wide variation in nasal width. Patients with maxillary atresia appear to have lower MCSA values. There was a tendency for MCSA measurements to increase (posterior nasal region) after ERMAC	MCSA – anterior region (pre-op) = 1.03 ± 0.23; MCSA – anterior region (post-op) = 1.05 ± 0.23; MCSA- posterior region (pre-op) = 1/24 ± 0.35; MCSA – posterior region (post-op = 1.56 ± 0.64
De Medeiros et al., 2017	Brazil	Prospective, single-centre, randomized, double-blind clinical trial	25 (19 female and 6 male). Mean age = 26.92 ± 2.919 years. SARME + PD = 12 patients; SARME − PD = 13 patients.	SARME + PD and SARME − PD	No	Volumetric measurements and area by CT	PD associated with SARME did not provide an increase in NCV, but did result in a significant volumetric expansion of the nasopharynx.	Nasal cavity volume: _PD (-175.4 mm^3^ ± 22); PD (494.3 mm^3^ ± 744.7); Volume of the nasopharynx: + PD (618.8 mm^3^ ± 120); =PD (351.6 mm^3^ ± 141.9).
Ferraro-Bezerra et al., 2018	Brazil	Prospective, single-centre, randomized, double-blind clinical trial	24 (18 females and 6 males). Mean age: 27.2 years)	SARME + PD and SARME − PD	No	Linear and angular measurements by CT	Linear increase in the floor of the nasal cavity, in the posterior region, after SARME + PD was greater than in the SARME − PD group.	Posterior region of the nasal floor SARME − PD (mean increase of 25%) SARME + PD (mean increase of 37%, although the Hyrax showed less expansion).
Kayalar et al., 2019	Turkey	Prospective clinical trial	20 (9 males	SARME + PD	No	CBCT	The mean piriform aperture width increased from 1.26 mm in T0-T1 to 0.97 mm in T1-T2 and 2.17 mm in T0-T2 (p< 0.008). In the soft tissue, the alar base width increased to 2.78 mm and the alar width to 2.95 mm in T0-T2 (p<0.001).	There was a positive correlation (63.6%) between the changes in skeletal and soft-tissue values.
Mitsuda et al., 2010	Brazil	Prospective clinical trial	27 (11 males and 16 females). Mean age: 28.03 years)	SARME + PD	No (measurements of all the patients with and without nasal decongestant)	AR	There was a statistically significant increase in MCSA and in VCN on the right and left, 6 months after SARME + PD, with and without the use of nasal decongestant. With the use of nasal decongestant, the values were higher than the means without decongestant.	Pre-/post-op percentage. Mean volume left with decongestant = 64.94; volume left without decongestant = 58.56 (p < .001).
Nada et al., 2013	Netherlands	Prospective cohort study	22 (11 male and 21 female). In the hyrax group: 19 patients (5 men and 14 women); TPD group: 3 patients (6 men and 7 women). With a mean age of 24.2 ± 7.0 years.	SARME + PD	No Hyrax group and TPD group).	Volumetric volume by CT and 3D photos.	Nasal volume in the soft tissues (nose) and nasal cavity volume.	Nasal volume in the soft tissues (nose) increased 1.01 ± 1.6% in the Hyrax group and 2.39 ± 2.4% in the TPD group (P = 0.008). The width of the nose wing increased in both groups between T0 and T1 (mean increase of 1.2 ± 0.9 mm for the Hyrax group and 1.4 ± 1.5 for the TPD group. There was no significant difference between the two treatment groups (p = 0.7). After 22 months, the nasal cavity volume increased by 9.7 ± 5.6% in the Hyrax group and 12.9 ± 12.7% in the TPD group.
Shwarz et al., 1985	USA	Retrospective study (not cited in the text)	9 (6 female and 3 male). Mean age = 28.6 years.	SARME + PD	No	Linear measurements and measurements of area by CT.	There were no significant linear changes in the position of the nasal septum before and after surgery. Therefore, the surgical cutting of the nasal septum to avoid septal deviation by SARME is not justified. Significant increases in the area of the available spaces in the nasal airways were recorded, due to the shrinkage of the inflamed nasal mucosa.	There was no change in the position of the nasal septum (p < 0.25). There was an increase in area of the nasal airspace (p < 0.01) after SARME + PD
Sygouros et al., 2014	Turkey	Retrospective study	20 (4 male and 16 female). Mean age = 18.8 years.	SARME + PD	No	Linear and angular measurements by CT	Shifting of the nasal floor in the direction of the occlusal plane.	Pyriform opening diameter increased in both groups (+ PD and − PD) with p = 0.001)
Zandi et al., 2016	Iran	Prospective study	30 (13 male and 17 female), with (15 of this study and 15 participants from another study). Mean age 21.26 years	SARME + PD and SARME − PD	No	Linear measurements by CT	Greater widening occurred at the level of the dental arch, compared with the nasal floor area, producing a "V"-shaped maxillary expansion in the coronal plane. There was no significant difference between the − PD and + PD groups.	Mean differences in nasal floor measurements between the groups. + PD group (Level of the first pre-molar = 1.53 ± 0.74; Level of the first molar – 12.47 ± 0.64). − PD group (level of the first pre-molar = 1.17 ± 0.59; level of the first molar = 1.17 ± 0.36).
Zawislak et al., 2020	Poland	Prospective study	36 (20 male and 16 female), with mean age of 27.1 years	SARME + PD	No	Frontal posteroanterior cephalogram	Craniofacial skeletal changes in adults with maxillary constriction after transpalatal distraction.	All patients reported improved nsasl patency after treatment. The smallest amount of width increase occurred at the nasal base.

SARME – Surgically-assisted rapid maxillary expansion; − PD = without pterygopalatine disjunction; + PD = With pterygopalatine disjunction; TPD = Transpalatal distractor; T0 = Preoperative period; T1 = Evaluation after the termination of active expansion (varied among the studies).

**Table 2:** Characterization of the studies included in relation to distraction and evaluation periods.

Authors	Type of distractor	Measuring periods	Rates of distraction	Latency period	Total amount of distraction (mean)
Baraldi et al., 2007	Tooth-borne (Hydrax) and dento-muco-supported (Haas)	Before treatment and 8.2 ± 2.7 months after the end of active expansion.	0.5 mm/day (0.25 in the morning and 0.25 at night	4 days	Not cited
De Medeiros et al., 2017	Tooth-borne (Hydrax)	Preoperative, immediately after stabilization of the Hyrax screw, and 6 months after the end of active expansion.	0.5 mm/day (0.25 in the morning and 0.25 at night	6 days	Not cited
Ferraro-Bezerra et al., 2018	Tooth-borne (Hydrax)	T0 (preoperative), T1 (end of expansion), and T2 (6 months after the final activation and removal of the Hyrax).	0.5 mm/day (0.25 in the morning and 0.25 at night	6 days	Measured in the Hyrax. SARME + PD = 6.2 ± 0.4 mm. SARME − PD = 5.8 ± 0.4 mm. The difference was not statistically significant.
Kayalar et al., 2019	Tooth-borne (Hyrax) and Tooth/bone-borne (hybrid Hyrax)	Preoperatively (T0), at the end of the active expansion phase (T1), and after 6 months of retention (T2)	0.5 mm/day (0.25 in the morning and 0.25 at night	14 days	Not cited
Mitsuda et al., 2010	Tooth-borne (Hydrax)	Before the treatment and around 6 months after the end of active expansion	0.5 mm/day (0.25 in the morning and 0.25 at night	Not cited	Not cited
Nada et al., 2013	Tooth-borne (Hydrax) and bone-borne (TPD)	Before the treatment and 22 ± 7 months after the end of active expansion.	1 mm/day	7 days	Not cited. At the level of the first molars, in the Hyrax group, the amount of bone expansion was 5.46 ± 3.3 mm, and in the TPD group, it was 3.4 ± 2.5 mm (not statistically significant, P = 0.13).
Shwarz et al., 1985	Not cited	Before the operation and around 4 months after the end of active expansion	0.5 mm/day (0.25 in the morning and 0.25 at night	Not cited	Not cited
Sygouros et al., 2014	Tooth-borne (Hydrax)	Before the operation and 3 and 6 months after the end of active expansion.	0.5 mm/day (0.25 in the morning and 0.25 at night	3 days	Not cited
Zandi et al., 2016	Tooth-borne (Hydrax)	Before the treatment and around 4 months after the end of active expansion,	0.5–0.6 mm/day	Not cited	SARME − PD = 7.9 ± 2.6 mm. SARME + PD = 7.3 ± 2.2 mm. The difference was not statistically significant.
Zawislak et al., 2020	Transpalatal distractor	Before treatment (T1) and after the completion of active distraction (T2)	0.5 mm/day (0.25 in the morning and 0.25 at night	7 days	Not cited

SARME – Surgically-assisted rapid maxillary expansion; − PD = without pterygopalatine disjunction; + PD = With pterygopalatinedisjunction; TPD = Transpalatal distractor; T0 = Preoperative period; T1 = Evaluation after the termination of active expansion (varied among the studies).

## Data Availability

The datasets used and/or analyzed during the current study are available from the corresponding author.

## References

[1] Betts NJ (2016). Surgically Assisted Maxillary Expansion. Atlas Oral Maxillofac Surg Clin North Am.

[2] Aloise AC, Pereira MD, Hino CT, Filho AG, Ferreira LM (2007). Stability of the transverse dimension of the maxilla after surgically assisted rapid expansion. J Craniofac Surg.

[3] Piccini A, Giorgetti R, Fiorelli G (1989). Stenosi respiratoria nasale ed ipoplasia mascellare. Modificazioni dopo trattamento ortodôntico com espasione rápida palatale. Acta Otorhinol Ital.

[4] Corey JP, Houser SM, Ng BA (2000). Nasal congestion: a review of its etiology, evaluation, and treatment. Ear Nose Throat J.

[5] Hartgerink DV, Vig PS, Abbott DW (1987). The effect of rapid maxillary expansion on nasal airway resistance. Am J Orthod Dentofacial Orthop.

[6] Koudstaal MJ, Smeets JB, Kleinrensink GJ, Schulten AJ, van der Wal KG (2009). Relapse and stability of surgically assisted rapid maxillary expansion: an anatomic biomechanical study. J Oral Maxillofac Surg.

[7] Ferraro-Bezerra M, Tavares RN, de Medeiros JR, Nogueira AS, Avelar RL, Studart Soares EC (2018). Effects of Pterygomaxillary Separation on Skeletal and Dental Changes After Surgically Assisted Rapid Maxillary Expansion: A Single-Center, Double-Blind, Randomized Clinical Trial. J Oral Maxillofac Surg.

[8] de Medeiros JR, Bezerra MF, Costa FWG, Bezerra TP, Alencar CRA, Soares ECS (2017). Does pterygomaxillary disjunction in surgically assisted rapid maxillary expansion influence upper airway volume? A prospective study using Dolphin Imaging 3D. Int J Oral Maxillofac Surg.

[9] Baraldi CE, Pretto SM, Puricelli E (2007). Evaluation of surgically assisted maxillary expansion using acoustic rhinometry and postero-anterior cephalometry. Int J Oral Maxillofac Surg.

[10] Mitsuda ST, Pereira MD, Passos AP, Hino CT, Ferreira LM (2010). Effects of surgically assisted rapid maxillary expansion on nasal dimensions using acoustic rhinometry. Oral Surg Oral Med Oral Pathol Oral Radiol Endod.

[11] Nada RM, van Loon B, Schols JG, Maal TJ, de Koning MJ, Mostafa YA (2013). Volumetric changes of the nose and nasal airway 2 years after tooth-borne and bone-borne surgically assisted rapid maxillary expansion. Eur J Oral Sci.

[12] Schwarz GM, Thrash WJ, Byrd DL, Jacobs JD (1985). Tomographic assessment of nasal septal changes following surgical-orthodontic rapid maxillary expansion. Am J Orthod.

[13] Sygouros A, Motro M, Ugurlu F, Acar A (2014). Surgically assisted rapid maxillary expansion: cone-beam computed tomography evaluation of different surgical techniques and their effects on the maxillary dentoskeletal complex. Am J Orthod Dentofacial Orthop.

[14] Zandi M, Miresmaeili A, Heidari A, Lamei A (2016). The necessity of pterygomaxillary disjunction in surgically assisted rapid maxillary expansion: A short-term, double-blind, historical controlled clinical trial. J Craniomaxillofac Surg.

[15] Kayalar E, Schauseil M, Hellak A, Emekli U, Fıratlı S, Korbmacher-Steiner H (2019). Nasal soft- and hard-tissue changes following tooth-borne and hybrid surgically assisted rapid maxillary expansion: A randomized clinical cone-beam computed tomography study. J Craniomaxillofac Surg.

[16] Zawiślak E, Gerber H, Nowak R, Kubiak M (2020). Dental and Skeletal Changes after Transpalatal Distraction. Biomed Res Int.

[17] Buck LM, Dalci O, Darendeliler MA, Papadopoulou AK (2016). Effect of Surgically Assisted Rapid Maxillary Expansion on Upper Airway Volume: A Systematic Review. J Oral Maxillofac Surg.

[18] Camps-Perepérez I, Guijarro-Martínez R, Peiró-Guijarro MA, Hernández-Alfaro F (2017). The value of cone beam computed tomography imaging in surgically assisted rapid palatal expansion: a systematic review of the literature. Int J Oral Maxillofac Surg.

[19] Hamedi Sangsari A, Sadr-Eshkevari P, Al-Dam A, Friedrich RE, Freymiller E, Rashad A (2016). Surgically Assisted Rapid Palatomaxillary Expansion With or Without Pterygomaxillary Disjunction: A Systematic Review and Meta-Analysis. J Oral Maxillofac Surg.

[20] Carvalho FSR, Studart Soares EC, Barbosa DAF, Mouta AEA, Bezerra TMM, Ribeiro TR (2019). Does surgically assisted rapid maxillary expansion associated with pterygomaxillary disjunction result in changes in mandibular position? A PROSPERO-compliant systematic review of the literature. J Craniomaxillofac Surg.

[21] Seeberger R, Kater W, Davids R, Thiele OC (2010). Long term effects of surgically assisted rapid maxillary expansion without performing osteotomy of the pterygoid plates. J Craniomaxillofac Surg.

[22] Kunkel M, Ekert O, Wagner W (1999). Changes in the nasal airway by transverse distraction of the maxilla. Mund-, Kiefer- und Gesichtschirurgie : MKG.

[23] Menegat F, Monnazzi MS, Silva BN, de Moraes M, Gabrielli MA, Pereira-Filho VA (2015). Assessment of nasal obstruction symptoms using the NOSE scale after surgically assisted rapid maxillary expansion. Int J Oral Maxillofac Surg.

[24] Magnusson A, Bjerklin K, Nilsson P, Jönsson F, Marcusson A (2011). Nasal cavity size, airway resistance, and subjective sensation after surgically assisted rapid maxillary expansion: a prospective longitudinal study. Am J Orthod Dentofacial Orthop.

[25] Deeb W, Hansen L, Hotan T, Hietschold V, Harzer W, Tausche E (2010). Changes in nasal volume after surgically assisted bone-borne rapid maxillary expansion. Am J Orthod Dentofac Orthop.

[26] Koudstaal MJ, Poort LJ, van der Wal KG, Wolvius EB, Prahl-Andersen B, Schulten AJ (2005). Surgically assisted rapid maxillary expansion (SARME): a review of the literature. Int J Oral Maxillofac Surg.

[27] Wriedt S, Kunkel M, Zentner A, Wahlmann UW (2001). Surgically assisted rapid palatal expansion. An acoustic rhinometric, morphometric and sonographic investigation. J Orofac Orthop.

